# Grasping movements toward seen and handheld objects

**DOI:** 10.1038/s41598-018-38277-w

**Published:** 2019-03-06

**Authors:** Ivan Camponogara, Robert Volcic

**Affiliations:** Department of Psychology, New York University Abu Dhabi, Abu Dhabi, United Arab Emirates

## Abstract

Grasping movements are typically performed toward visually sensed objects. However, planning and execution of grasping movements can be supported also by haptic information when we grasp objects held in the other hand. In the present study we investigated this sensorimotor integration process by comparing grasping movements towards objects sensed through visual, haptic or visuo-haptic signals. When movements were based on haptic information only, hand preshaping was initiated earlier, the digits closed on the object more slowly, and the final phase was more cautious compared to movements based on only visual information. Importantly, the simultaneous availability of vision and haptics led to faster movements and to an overall decrease of the grip aperture. Our findings also show that each modality contributes to a different extent in different phases of the movement, with haptics being more crucial in the initial phases and vision being more important for the final on-line control. Thus, vision and haptics can be flexibly combined to optimize the execution of grasping movement.

## Introduction

In everyday life, actions are not only directed toward objects we see, but also toward objects we already hold in one hand. For instance, even without vision, we can easily grasp the lid of a marmalade jar, while holding the jar with the other hand. Thus, haptic information (i.e., proprioceptive inputs from tendon, muscle spindles and joint receptors in concert with tactile inputs) is sufficient to select and perform the proper action by specifying both the direction and amplitude of the movement as well as the preshaping of the hand. While the planning, execution and control of grasping movements under visual guidance have been extensively studied^1–7^, how humans perform grasping movements based on haptic input or on the combination of visual and haptic sensory inputs remains to be fully established.

Several studies have compared the use of visual and haptic information in *reaching* movements by asking participants to move one hand to a visual target or to their other unseen hand^8–16^. The end points of reaching movements based only on haptic information are generally more variable than those directed toward visual targets. However, even if haptic information might seem to be unreliable, it still plays an important role. When haptic information about the target position is complemented by the visual information, the end points of reaching movements are more accurate and more precise than in the conditions based on a single sensory modality, supporting the idea that multisensory information can be efficiently combined during sensorimotor processing^8,10,17–20^.

In contrast to reaching, grasping actions toward a haptically sensed object need to be based not only on extrinsic properties (i.e., object’s location), but also on intrinsic properties of the object (i.e., size)^21–26^. These properties can be acquired also through haptics. Proprioceptive inputs from the extended arm and from the fingers enclosing the object inform the central nervous system about the object location in space and about its size, respectively^27^. These inputs are enriched by the additional tactile inputs induced by different skin stretch patterns of the arm and the hand^28^. Haptic inputs are indeed sufficient to guide grasping movements^23,29^. For instance, the maximum grip aperture of the contralateral hand consistently scales with the haptically perceived size of the object. However, how does grasping under haptic guidance compare to visually guided grasping? And, does haptic information support vision when both are available during the execution of a grasping action?

Only a handful of studies have directly compared grasping actions under visual only and haptic only guidance^29,30^. When grasping was based only on haptic information, the maximum grip aperture was usually wider and movements took longer compared to vision. Surprisingly, when grasping was under combined visuo-haptic guidance, movements were very similar to those based on only visual information^30^ which raises the question what the reason may be that multisensory information is not exploited during grasping movements. One reason may be that vision provides us with sufficiently accurate and reliable information and thus grasping is already maximally optimized. A simpler reason may be that the haptic and visual information were considered as not belonging to the same object^31,32^ given that both the haptically-explored object and the hand feeling this object were hidden below a platform on which the seen object was attached^30^. Thus, haptic information may have been completely ignored and movements were planned and executed by relying on visual information alone.

The failure of observing multisensory integration in grasping movements is particularly surprising in light of the following considerations. Humans can effortlessly judge the size by enclosing an object between the digits^33–36^. In fact, haptic feedback obtained after grasping an object is considered to play an important role in calibrating goal directed actions^37–44^. Hence, blending the haptic with the visual information when both are available even prior to movement start would be the best strategy to minimize the risk of misestimating the position and/or the size of an object.

In the present study, we compared grasping actions under haptic only, visual only or visuo-haptic guidance to further investigate the unisensory and multisensory processes in prehension. In the visual condition, participants had full vision of the object and the workspace. In the haptic condition, vision was prevented and participants used their left hand to continuously feel the object that was the target of the grasping action performed with their right hand. Finally, in the visuo-haptic condition, participants could make use of both visual and haptic information throughout the movement. Importantly, in the visuo-haptic condition, the object that was the target of the grasping action was the exact same object that the participants felt with the other hand.

To understand how unisensory and multisensory information is used to plan and execute grasping movements we have monitored the movements of the hand and the digits as the reach and grasp unfolded throughout the entire movement trajectory instead of relying only on specific kinematic markers (maximum grip aperture, movement duration, etc.). There are important advantages of this approach. First, movements that are indistinguishable in terms of kinematic markers might turn out to fundamentally differ in other important aspects. And, second, the relative role of visual and haptic information in multisensory integration is not necessarily fixed and might instead dynamically develop along the movement.

## Methods

### Participants

We tested a sample of 20 participants (10 male, mean age 19.4), recruited at New York University Abu Dhabi. All had normal or corrected-to-normal vision and no known history of neurological disorders. All the participants were naïve to the purpose of the experiment and were provided with a subsistence allowance. The experiment was undertaken with the understanding and written informed consent of each participant and experimental procedures were approved by the Institutional Review Board of New York University Abu Dhabi in compliance with the Code of Ethical Principles for Medical Research Involving Human Subjects of the World Medical Association (Declaration of Helsinki).

### Apparatus

**Figure 1:**
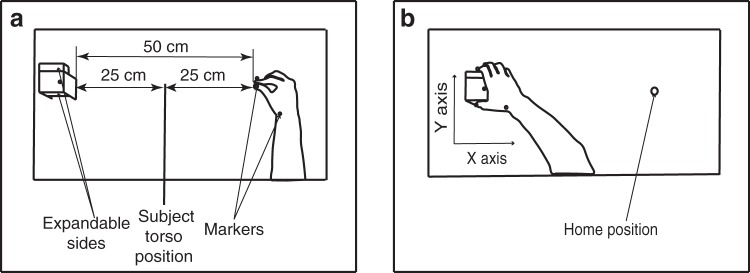
Experimental setup: top view of the subject’s start (**a**) and end (**b**) positions.

The stimulus consisted of a custom made wooden rectangular cuboid of 5 cm length, 4 cm width and 3 cm height, raised 10 cm above the table top. The two sides along its *y*-axis could slide out to change the size of the stimulus^45,46^ (see Fig. 1a) and were moved by two mini-pneumatic cylinders embedded in the object (Camozzi, model QP2A012A010, 4 cm long, 2.5 cm wide, 1 cm stroke). These pneumatic cylinders were linked to two solenoid valves (AirTac, 5/2 Way 4V210-06) which were connected to an air compressor. The solenoid valves were controlled by an Arduino Uno (Arduino 2016) via Matlab (Mathworks Inc, Natick, MA, USA). A command sent from Matlab to Arduino activated the selected solenoid valve which released the compressor’s air into the piston. The selected side then slid-out 20 ms after the initial command. The object was of 5 cm length when both sides were retracted, and 6 cm when one of the two sides was slid-out. The object was located at a distance of 50 cm along the *x*-axis from the home position (a 0.5 cm high rubber bump with a diameter of 0.9 cm attached to the table, Fig. 1b).

A pair of occlusion goggles (Red Scientific, Salt Lake City, UT, USA) was used to prevent vision of the workspace in the haptic condition and between trials. A pure tone of 1000 Hz, 100 ms length, 65 dB of intensity was used to signal the start of the trial, while another one of 600 Hz with the same length and intensity to signal its end.

Index digit, thumb and wrist movements were acquired on-line at 200 Hz with sub-millimeter resolution by using an Optotrak Certus system (Northern Digital Inc., Waterloo, Ontario, Canada). Markers were attached on the first phalanx of the thumb and index digit onto the, respectively, lateral and medial fingernail top side, and on the styloid process of the radius. The Optotrak system and the occlusion goggles were both controlled by a custom Matlab program.

### Procedure

Participants sat comfortably in front of the table, with the center of their torso positioned between the object, located on their left side, and the home position, located on their right side (Fig. 1a). Participants were required to perform a precision grip with their right thumb and index digit along the *y*-axis of the stimulus in three different conditions: Visual (V): Participants were allowed to see the object (goggles open); Haptic (H): Vision was prevented, participants were allowed to touch the object along its *y*-axis with the left hand (goggles closed); Visuo-haptic (VH): Participants were allowed to both see and touch the object (goggles open).

All the trials started with the participants’ thumb and index digit of the right hand positioned on the home position (Fig. 1a), the left hand positioned on the left side of the object (at a comfortable distance) and the shutter goggles closed. Before each trial the object was set to the appropriate size (5 cm or 6 cm), and: 1) in the H condition, the experimenter signaled to the participants to touch the object with their left hand (i.e., sense its size and position by means of tactile and proprioceptive feedback) while shutter goggles remained closed, or, 2) in the V condition, the goggles turned transparent to enable the participant to see the object, or, 3) in the VH condition, the participants had to touch the object with their left hand and the goggles turned transparent.

**Figure 2:**
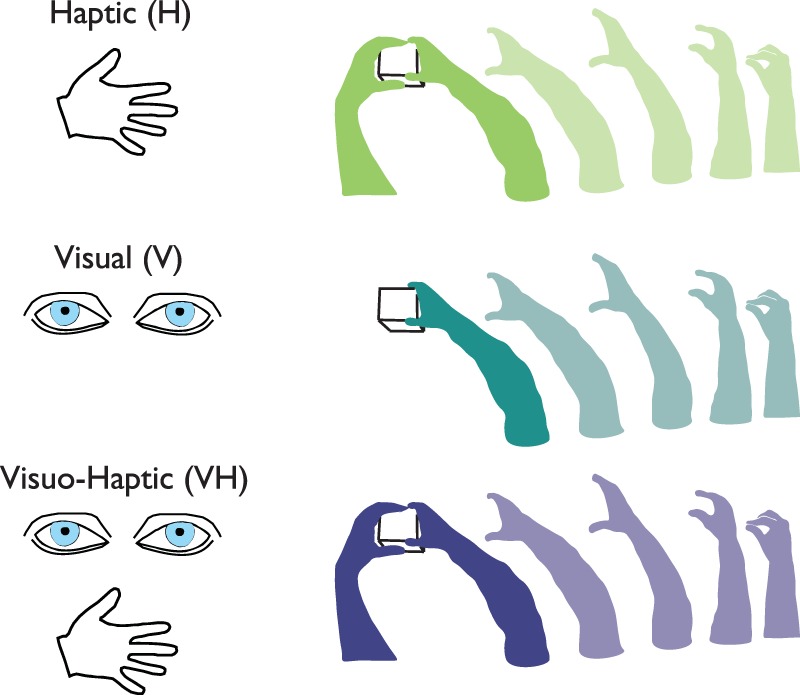
Representation of the task under Haptic, Visual and Visuo-Haptic condition. The grasping action was always performed with the right hand. In H and VH participants were already holding the object with their left hand before the start of the grasping action.

After a variable period, the start tone was delivered and participants had to perform a reaching and grasping action at their natural speed towards the object (Fig. 1b). No reaction time constrains were imposed. After 3 seconds the end sound was delivered, and, only in the H modality, the goggles were made transparent. Participants had to move their right hand back to the home position, the left one on the object’s side and then the goggles turned opaque. The object was then set to a new configuration and another trial started (the task under each condition is schematically represented in Fig. 2).

To introduce some variability in the task, three different configurations of the cube’s sides were used: one where both the expandable sides were in, one where the thumb side was slid out and one where the index digit side was slid out. All the analyses will consider the latter two configurations in which the object was of the same size (6 cm). These data were collapsed for all further analyses. Each modality condition was recorded in a separate block of trials. The order of these blocks was randomized across participants, while sides configurations were randomized within each modality. We ran ten trials for each configuration which led to a total of 90 trials per participant (30 for each modality). Before the experiment, a training session was performed in which ten trials were run in each modality to accustom the participants with the task.

### Data analysis

Kinematic data were analyzed in R^47^. The raw data were smoothed and differentiated with a third-order Savitzky-Golay filter with a window size of 21 points. These filtered data were then used to compute velocities and accelerations in three-dimensional space for each digit and the wrist. Movement onset was defined as the moment of the lowest, non-repeating wrist acceleration value prior to the continuously increasing wrist acceleration values^48^, while the end of the grasping movement was defined on the basis of the Multiple Sources of Information method^49^. We used the criteria that the grip aperture is close to the size of the object, that the grip aperture is decreasing, that the second derivative of the grip aperture is positive, and that the velocities of the wrist, thumb and index digits are low. Moreover, the probability of a moment being the end of the movement decreased over time to capture the first instance in which the above criteria were met^48^.

Trials in which the end of the movement was not captured correctly or the missing marker samples could not be reconstructed using interpolation were discarded from further analysis. The vast majority of the trials with missing marker samples was among the same four participants. The exclusion of these participants left us with 960 trials from which another 61 trials had to be removed from further analysis.

#### Kinematic markers analysis

**Figure 3:**
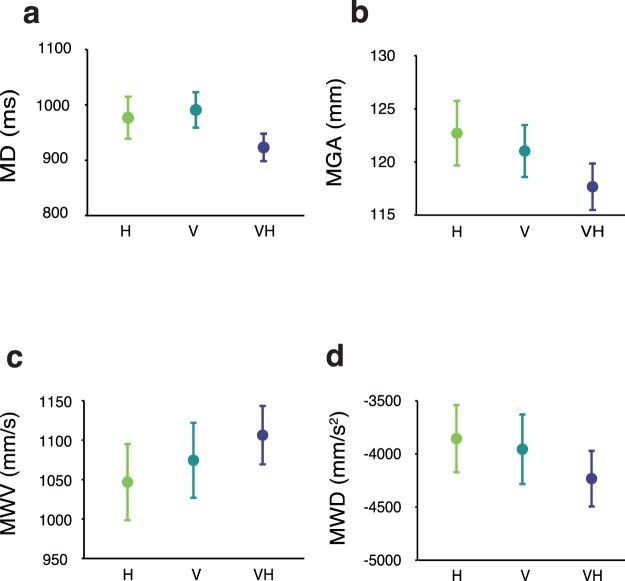
Average performance in terms of (**a**) movement duration, (**b**) maximum grip aperture, (**c**) maximum wrist velocity, (**d**) maximum wrist deceleration, in the haptic (H), visual (V), and visuo-haptic (VH) conditions. Error bars represent the standard error of the mean.

**Figure 4:**
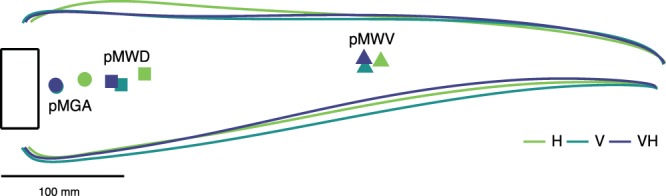
Top view of the average thumb and index digit trajectories in the three conditions. The movement unfolded from right to left. The lines on the top represent the trajectories of the index digit, the lines on the bottom represent the trajectories of the thumb. H, V and VH modalities are represented respectively in light green, dark green and blue. Triangles represent the position along the trajectory where participants reached the MVW, squares the MWD and circles the MGA. The vertical position of the symbols coincides with the midpoint between the thumb and index digit positions. The pMWV, pMWD and pMGA occurred at 0.45 ± 0.04, 0.81 ± 0.03 and 0.90 ± 0.02 in H, at 0.47 ± 0.04, 0.84 ± 0.03, and 0.94 ± 0.01 in V, and, in 0.48 ± 0.04, 0.86 ± 0.02, and 0.94 ± 0.01 in VH. The pMGA of the V condition is not visible, because it is occluded by the VH symbol.

For each trial, we calculated the movement duration (MD), defined as the time from the movement onset to the end of the trial, the maximum grip aperture (MGA), defined as the maximum Euclidean distance between the thumb and the index digits, the maximum wrist velocity (MWV) and the maximum wrist deceleration (MWD) (Fig. 3). Moreover, we calculated the point of the trajectory at which the maximum grip aperture (pMGA), the maximum wrist velocity (pMWV) and the maximum wrist deceleration (pMWD) occurred (Fig. 4).

For each kinematic variable we performed a repeated measures ANOVA considering as within factor the three modalities: Visual, Haptic and Visuo-haptic. Bonferroni correction was applied to the follow-up pair-wise comparisons.

#### Space-normalized trajectories

To analyze how the movements of the hand and the digits unfolded throughout the movement, we have spatially normalized each grasping movement using the total length of the movement, i.e., the length of the trajectory that the midpoint between the digits covered from the starting position to the end of the movement^48^. Each wrist, thumb and index digit trajectory was resampled in 201 points evenly spaced along the three-dimensional trajectory in the range from 0 (movement onset) to 1 (movement end) in 0.005 steps using cubic spline interpolation.

**Figure 5:**
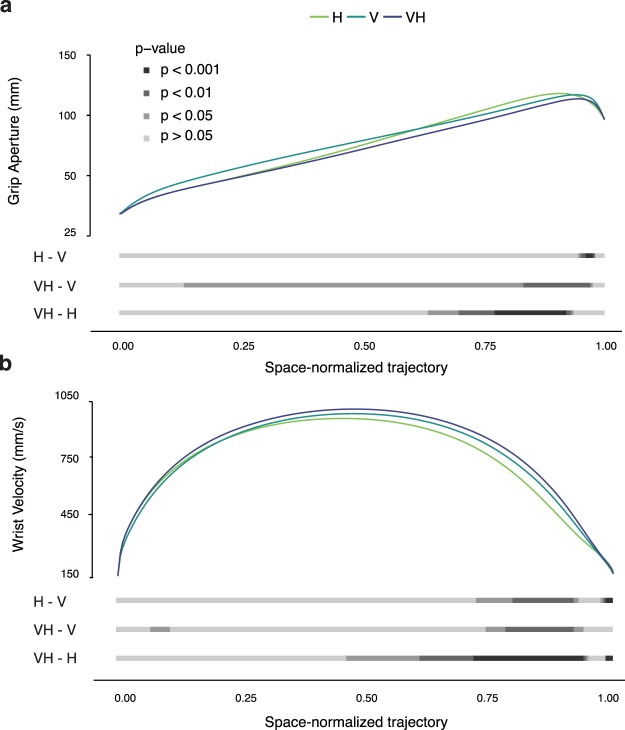
Average (**a**) grip aperture and (**b**) wrist velocity in the H, V and VH conditions along the space-normalized trajectory. Shaded horizontal bars below each panel represent the evolution of the *p*-value along the trajectory for each comparison.

Grip aperture and wrist velocity were then determined at each step of the space-normalized trajectories. To investigate the unfolding of the grip aperture and the wrist velocity between modalities (H-V, VH-V, and VH-H) we ran a Bonferroni corrected paired *t*-tests at each step of the space-normalized trajectories (Fig. 5).

## Results

### Kinematic markers

Table 1 reports the results of the statistical analyses on the kinematic markers. In general, we found a similar MD, MGA, MWV, and MWD in H and V modalities (Fig. 3). Instead, the VH-V and VH-H comparisons showed that participants had a shorter MD in VH compared to V (Fig. 3a) and a smaller MGA in VH compared to each unisensory conditions (Fig. 3b). Moreover, we also observed higher wrist velocities in VH compared to H (Fig. 3c). These advantages suggest that movements were influenced by the available sensory information already prior to the start of the movement: having both visual and haptic information about the target object led to faster movements^50–53^. Interestingly, this movement rapidity did not hinder performance, quite the contrary, the MGA reduction suggests that the joint visuo-haptic information actually optimized grasping movements.

**Table 1:** Kinematic markers results.

Variable	Main effect		H-V		VH-V		VH-H	
	*F*(2,30)	*p*	*t*(15)	*p*	*t*(15)	*p*	*t*(15)	*p*
MD	5.61	0.008	−0.60	1	−3.83	0.004	−2.37	0.09
MGA	8.38	0.001	0.93	1	−3.97	0.003	−3.94	0.003
MWV	3.35	0.04	−1.10	0.83	1.38	0.55	2.71	0.04
MWD	3.42	0.04	0.46	1	−1.87	0.23	−2.57	0.06
pMGA	32.63	<0.001	−5.30	<0.001	2.32	0.1	6.50	<0.001
pMWV	7.37	0.002	−2.59	0.06	0.45	0.65	3.90	0.004
pMWD	17.97	<0.001	−2.68	0.05	2.76	0.04	8.05	<0.001

The rows list the following dependent variables: maximum grip aperture (MGA), maximum wrist velocity (MWV), maximum wrist deceleration (MWD), the proportion of the movement trajectory at which MGA (pMGA), MWV (pMWV), and MWD (pMWD) occurred. The Main effect column represents the outcome of a repeated measures ANOVA. The H-V, VH-V, and VH-H columns represent the outcomes of pairwise comparisons (Bonferroni corrected) between H, V, and VH conditions.

The above analyses did not show striking differences between the H and V modalities. However, based on this evidence we cannot conclude that the actions were actually performed in a similar fashion. In fact, the analysis of pMGA and pMWD showed that the MGA and MWD occurred earlier along the movement in H compared to V and VH (Fig. 4). On the other hand, the kinematic peaks in V and VH occurred in virtually the same portions of the movement. It is clear that the available sensory information directly determined the planning and execution of grasping movements. To investigate these distinctions more thoroughly we proceeded with a series of analyses that take into account the grip aperture and the wrist velocity along the whole unfolding of the movement (i.e., along the space-normalized trajectory).

### Space-normalized trajectories

#### Grip aperture

The comparison between H an V modalities showed that under visual guidance the grip aperture was significantly smaller only from the 96% to the 99.5% of the trajectory (Fig. 5a, H-V). When both vision and haptics were available, the grip aperture was smaller for large parts of the trajectory compared to each single modality. In particular, we found a significantly smaller grip aperture in VH compared to V modality from the 13% to the 98.5% of the trajectory. Interestingly, the comparison between VH and H modality showed an indistinguishable grip aperture for the first part of the movement and a significantly reduced grip aperture in VH only later on, from the 64.5% to the 94.5% of the trajectory (Fig. 5b, VH-V, VH-H). The haptic modality thus seems to have an important role in action guidance in the early stages of the action, whereas vision takes control in the later stages, when the hand is in the object’s proximity.

#### Wrist velocity

The wrist velocity in H was significantly lower than in V from the 73% to the 94% (and for the last 1%) of the trajectory (Fig. 5b, H-V) and it was also significantly lower than in VH from the 47% to the 96% (and for the last 1%) of the trajectory (Fig. 5b VH-H). In contrast, the wrist velocity in V was significantly lower than in VH from the 7% to the 10.5%, and a second time from the 75% to the 95% of the trajectory (5b, VH-V). Again, as we have found in the grip aperture analysis, our data suggest that vision plays a very relevant role in the final phases of the movement. However, the faster movement in VH compared to V supports the idea that visual and haptic information is combined to accomplish the grasping action in the most efficient way.

## Discussion

The aims of the present study were twofold. First, we compared grasping movements toward an object that was only seen (visual input) and toward an object that was continuously felt by the other non-grasping hand (haptic input based on both proprioception and touch) to unravel the differences and the similarities between these grasping categories. Second, we tested whether the motor system makes use of the multisensory information when grasping movements are under both haptic and visual guidance. Our results show that grasping movements under haptic or visual guidance share some similarities but also exhibit some critical differences. In addition, the combined visuo-haptic information optimizes grasping by speeding up the movement and, at the same time, by reducing the grip aperture that is necessary to successfully grasp the object.

The similarities based on kinematic parameters (MD, MGA, MWD and MWV) between haptically- and visually-guided grasping might suggest that haptics is as good as vision in providing the motor system with reliable information about the position and shape of the object. But this was not the case. In the H condition, the main kinematic parameters (pMGA, pMWV and pMWD) occurred earlier along the trajectory of the movement than in V. This pattern is even more explicit when we look at how the grasping actions under H or V evolved along the whole trajectory. These differences indicate that, in H, hand preshaping was initiated earlier, the digits closed on the object more slowly, and the final phase was more cautious, most likely, to keep a sufficiently wide safety margin to avoid an accidental collision with the object^29,30^. Wider grip apertures are normally found when vision of the hand is prevented^54–60^, but, interestingly, the same strategy was also used in H, even though the haptic on-line feedback from the non-grasping hand was potentially available. From this we may infer that haptic information about either the size or the position of the object, or both, is less reliable than in vision and it thus promotes a different grasping behavior.

When actions were under both haptic and visual guidance, we observed a very different grasping behavior, which showed clear evidence of visuo-haptic integration. The grasping actions in the VH condition benefited from multisensory inputs in several ways: movements were performed in a shorter amount of time compared to the V condition, and the grip opened less than in H and V conditions. These results are at odds with those found by Pettypiece and colleagues^30^ who observed a similar MGA in V and VH conditions. A possible explanation could be that visual and haptic signals were not combined because they were treated as coming from two different objects; a strong evidence against the unity assumption^31,32^. Instead, participants in our study could see that their left hand was holding the object while performing the reach-to-grasp action with the right hand, thus making it obvious that what they were feeling was the same object that they were looking at.

Interestingly, haptics and vision seem to each play different roles along the movement trajectory. Whereas the VH grip aperture started to differ from V already in the initial phases of the movement, the differences between VH and H appeared only later. On the contrary, the differences in terms of wrist velocity arose earlier between H and VH, than between V and VH conditions. The advantage of the VH condition with regards to the unisensory conditions seems to rely on a more predominate role of H in the initial phases which gives place to V in the final phases when on-line visual feedback becomes more crucial^48^.

Taken together, these results reveal a clear advantage of visuo-haptically guided grasping. However, one open question concerns the contribution of the different haptic and visual signals. Visual estimates of size are tightly linked to visual estimates of distance: we can properly scale retinal information only if our visual distance estimates are approximately correct^61–67^. On the other hand, two signals supply us with independent haptic information. The fingers enclosing the object provide information about the object size, and, the flexion-extension of the arm provides information about the position of the object. Thus, the visual and haptic signals could be integrated in several ways to plan the grasping action.

In the case of full integration, the haptic position estimate would be integrated with the visual position estimate to promote a better visual size estimate which, in turn, would be integrated with the haptic size estimate. Instead, in the case of partial integration, two options exist. In one case, the estimate of distance would be based only on vision and thus only the haptic size estimate would be integrated with the visual size estimate. In another case, only the haptic and visual distance estimates would be integrated to provide a multisensory distance estimate used to scale the visual size information. In addition, once the movement has started, the sensory feedback from the visual and haptic modalities needs to be integrated also in real-time along the movement trajectory. The current data do not allow to distinguish between these scenarios and they will thus needed to be addressed in future studies.

In summary, we showed that the simultaneous availability of both visual and haptic information of the target object substantially improves reach-to-grasp actions. Thus, the nervous system combines visual and haptic information to optimize movement execution.

## Data Availability

The authors can make the data available upon request.
